# Factors associated with acceptance of COVID-19 vaccine among University health sciences students in Northwest Nigeria

**DOI:** 10.1371/journal.pone.0260672

**Published:** 2021-11-29

**Authors:** Mohammed Mustapha, Basira Kankia Lawal, Abubakar Sha’aban, Abubakar Ibrahim Jatau, Abubakar Sadiq Wada, Auwal Adam Bala, Sagir Mustapha, Anas Haruna, Abbas Musa, Mubarak Hussaini Ahmad, Salim Iliyasu, Surajuddeen Muhammad, Fatima Zaji Mohammed, Ahmed Danbala Ahmed, Hadzliana Zainal

**Affiliations:** 1 School of Pharmaceutical Sciences, Universiti Sains Malaysia, Minden, Pulau Pinang, Malaysia; 2 Faculty of Pharmaceutical Sciences, Department of Clinical Pharmacy and Pharmacy Practice, Ahmadu Bello University, Zaria, Kaduna, Nigeria; 3 Faculty of Pharmaceutical Sciences, Department of Clinical Pharmacy and Pharmacy Management, Kaduna State University, Kaduna, Nigeria; 4 School of Pharmacy and Pharmacology, University of Tasmania, Hobart, Australia; 5 Department of Pharmacology and Therapeutics, Bayero University Kano, Kano, Nigeria; 6 Department of Pharmacology, College of Medicine and Health Sciences, Federal University Dutse, Jigawa, Nigeria; 7 Department of Pharmacology, School of Medical Sciences, Universiti Sains Malaysia, Kubang Kerian, Kelantan, Malaysia; 8 Faculty of Pharmaceutical Sciences, Department of Pharmacology and Therapeutics, Ahmadu Bello University, Zaria, Kaduna, Nigeria; 9 Faculty of Pharmaceutical Sciences, Department of Pharmaceutical and Medicinal Chemistry, Kaduna State University, Kaduna, Nigeria; 10 Department of Pharmaceutics and PharmaceuticalTechnology, Bayero University Kano, Kano, Nigeria; 11 Faculty of Veterinary Medicine, Ahmadu Bello University, Zaria, Kaduna, Nigeria; 12 School of Dental Health Sciences, Shehu Idris Institute of Health Sciences and Technology, Kaduna State University, Makarfi, Kaduna, Nigeria; 13 Faculty of Pharmaceutical Sciences, Department of Pharmacology and Toxicology, Kaduna State University, Kaduna, Nigeria; University of South Carolina College of Pharmacy, UNITED STATES

## Abstract

Students of the health sciences are the future frontliners to fight pandemics. The students’ participation in COVID-19 response varies across countries and are mostly for educational purposes. Understanding the determinants of COVID-19 vaccine acceptability is necessary for a successful vaccination program. This study aimed to investigate the factors associated with COVID-19 vaccine acceptance among health sciences students in Northwest Nigeria. The study was an online self-administered cross-sectional study involving a survey among students of health sciences in some selected universities in Northwest Nigeria. The survey collected pertinent data from the students, including socio-demographic characteristics, risk perception for COVID-19, and willingness to accept the COVID-19 vaccine. Multiple logistic regression was used to determine the predictors of COVID-19 vaccine acceptance. A total of 440 responses with a median (interquartile range) age of 23 (4.0) years were included in the study. The prevalence of COVID-19 vaccine acceptance was 40.0%. Factors that independently predict acceptance of the vaccine were age of 25 years and above (adjusted odds ratio, aOR, 2.72; 95% confidence interval, CI, 1.44–5.16; *p* = 0.002), instructions from heads of institutions (aOR, 11.71; 95% CI, 5.91–23.20; *p*<0.001), trust in the government (aOR, 20.52; 95% CI, 8.18–51.51; *p*<0.001) and willingness to pay for the vaccine (aOR, 7.92; 95% CI, 2.63–23.85; *p*<0.001). The prevalence of COVID-19 vaccine acceptance among students of health sciences was low. Older age, mandate by heads of the institution, trust in the government and readiness to pay for the vaccine were associated with acceptance of the vaccine. Therefore, stakeholders should prioritize strategies that would maximize the vaccination uptake.

## 1.0 Introduction

The novel coronavirus disease 2019 (COVID-19), caused by severe acute respiratory syndrome coronavirus 2 (SARS-CoV-2), has become a major public health problem since the initial outbreak in Hubei, Wuhan, China [1]. The pandemic has significantly impacted health systems and economies globally [2–4]. The pandemic is one of the fatal outbreaks due to a coronavirus, including the severe acute respiratory syndrome (SARS) in 2003 [5] and the Middle East respiratory syndrome (MERS) in 2012 [6]. Countries with weaker health systems, particularly those in Africa, were projected to be devastated by the COVID-19 [7]. Thus, there is a need for an effective regional response that prioritize strategies for protecting all vulnerable population.

Following the sequencing of the SARS-CoV-2 genome, there has been rapid development of COVID-19 vaccine candidates. Several vaccines are presently at different phases of clinical evaluation. In addition, some of the vaccines have since obtained Emergency Use Authorization (EUA) from the World Health Organization (WHO) and other relevant authorities [8,9]; notably, BioNTech/Pfizer® (BNT162b), Moderna® (mRNA-1273), Janssen® (Ad26.COV2.S), AstraZeneca® (ChAdOx1 nCoV-19) vaccines and host of others. Since vaccination coverage is a defining factor for successful herds immunity, there is a need to explore the determinants of COVID-19 vaccine uptake that could guide effective vaccination strategies.

The National Agency for Food and Drug Administration and Control (NAFDAC) of Nigeria has approved the use of the AstraZeneca® (ChAdOx1 nCoV-19) vaccine for use in the country [10], and presently several others. The Nigerian government has received several batches of the vaccine via the COVID-19 Vaccines Global Access Facility (COVAX), a partnership between the Coalition for Epidemic Preparedness Innovations (CEPI), Gavi the Vaccine Alliance, the United Nations Children’s Fund (UNICEF), the World Bank, and the WHO. The COVAX is part of the Access to COVID-19 Tools (ACT) Accelerator, a global collaboration to accelerate development, production, and equitable access to tests, treatments, and vaccines for COVID-19 [11]. Nigeria’s National Primary Health Care Development Agency (NPHCDA) has since commenced stagewise vaccination with priority groups, starting with frontline healthcare workers. The agency has also deployed a self-registration portal online to roll out a country-wide vaccination program.

Despite the wide availability of the COVID-19 vaccine, previous experience suggests huge limitations on vaccination uptake [12,13]. Sometimes, available information on the COVID-19 are misleading, resulting in confusion and overload [14]. The unfortunate experiences from the previous pandemics (e.g., Influenza), particularly the low acceptance in many countries, suggest the need for an improved understanding of the vaccine’s uptake and hesitancy [15–17]. The World Health Organization (WHO) Strategic Advisory Group of Experts on Immunization (SAGE) has described vaccine hesitancy as "the delay in acceptance or refusal of vaccination despite the availability of vaccination services" [18]. Vaccine hesitancy is considered a significant threat to global health, and thus, due to regional and population variability, there is a need to explore local data to add to the global data pool on COVID-19 vaccine acceptance and hesitancy.

Several studies from different parts of the world have examined the acceptance of the COVID-19 vaccine in a critical group like young adults, including students of tertiary institutions and the healthcare profession [19–22]. The students’ participation in COVID-19 response varies across countries and primarily for educational purposes [23,24]. However, the role of healthcare students, particularly the medical students, in COVID-19 response programs is evolving rapidly due to the shortage of healthcare professionals in many countries [24,25]. There are recommendations that the medical students can work as volunteers, undergo appropriate training, limit activity within their level of competence, and receive continuous supervision in the course of COVID-19 response [25]. Moreover, they also provide advocacy and recommendations to the public regarding disease education and prevention. The health literacy of health sciences students could be influenced by several socioeconomic factors, including gender, age, household income, the field and level of study, and history of chronic disease [26]. Thus, the present study aimed to evaluate the factors associated with COVID-19 vaccine acceptance among health sciences students in Northwest Nigeria.

## 2.0 Methods

### 2.1 Study design

The study was a cross-sectional study involving an open online self-administered survey via Google Forms (www.google.com/forms). The participants were invited by sharing the hyperlink to the survey through emails, social media (Facebook, Twitter and WhatsApp) pages, and groups. Reminders were sent occasionally to improve the response rate. The survey data were collected between 15 March 2021 and 14 June 2021. A convenience sampling with a simplified snowball sampling technique was used to recruit the participants for this study. The invited participants were requested to post the study invitations on their social media pages and groups. The Google Form was pre-set to limit to only one response using their unique email address. Participants with complete and valid responses were included in the final analysis. The completion rate of the survey was determined by dividing the number of participants who completed the survey by the total number who responded. Based on a previous study, participants with careless responses were identified and excluded from the study [27]. The survey was conducted and reported based on the Strengthening the Reporting of Observational Studies in Epidemiology (STROBE) guidelines (S1 File) [28].

### 2.2 Study setting and participants

The study was conducted among health sciences students from four selected tertiary institutions (Ahmadu Bello University Zaria, Kaduna, Bayero University Kano, Federal University Dutse, Jigawa, and Kaduna State University, Kaduna) in North-western Nigeria. Northern Nigeria is the most populous and diverse region in Nigeria, constituting nineteen out of the thirty-six states in the country, including the Federal Capital Territory, Abuja. The indigenous people of the region are mainly Hausa and Fulani by tribe. Other ethnic groups from different parts of the country are also found primarily in the city centres that host the institutions used in this study. The study included students of Medicine, Pharmacy, Nursing and Other health-related courses (Such as Radiology, Optometry and Dentistry) aged 18 years and above.

### 2.3 Survey tool and outcome measures

The study survey tool was developed based on a validated vaccine hesitancy scale provided by the World Health Organization (WHO) Strategic Advisory Group of Experts on Immunization (SAGE) [29,30], relevant literature search and discussion with experts. The final survey tool was reviewed, and content validated by three independent experts. The tool was pilot tested on ten university health science students for face validity. The measure of internal consistency reliability of the survey tool, Cronbach’s alpha, was found to be 0.88. The sample used for the pilot was excluded from the main study.

The study survey tool (S2 File) consists of three main sections. The first section collected socio-demographic data (gender, age, marital status, family income, course, level of study, history of chronic disease). The second section collected information on COVID-19 risk perception (experiences with COVID-19, previous vaccination history. The final section collected data on the acceptability of a COVID-19 vaccine (willingness to receive or pay for the COVID-19 vaccine, trust in government, influence of heads of institutions and vaccine recommendation). All questions were close-ended with categorical variables except for age (years). The acceptance of the COVID-19 vaccine was assessed as a dichotomized option: acceptance (yes = 1, will take the vaccine) and refusal (no = 0, will not take the vaccine). The tool was provided in English since the students were familiar with the language.

### 2.4 Sample size determination

The minimum sample size for the study was calculated using the Epi-Info™ 7.2.6.0 software for windows (Center for Disease Control and Prevention (CDC), Atlanta, Georgia, USA).

Sample size, n = [DEFF*Np(1-p)]/[(d^2^/Z^2^_1-α/2_*(N-1)+p*(1-p)] = 382

Population size (N): 15,000; Hypothesized (%) frequency of outcome in the population (p): 50% +/-5; Confidence limits as % of 100 (absolute +/%) (d): 5%; Design effect (DEFF): 1; Z is a constant = 1.96. for 95% confidence interval (CI).

The minimum required sample size required for our study was 382 health sciences, students.

### 2.5 Data preparation and analysis

The survey responses from the Google Forms were downloaded into a Microsoft Excel spreadsheet and then imported into IBM SPSS Statistics for Windows, version 26.0 (IBM Corp., Armonk, NY, USA.) for data analysis. Missing data were handled using multiple imputation methods [31]. A normality test was conducted on the continuous numerical variables using the histogram and Kolmogorov-Smirnov test. Data were reported using descriptive statistics. Categorical variables were presented as frequencies and percentages, and continuous variables as mean (standard deviation) or median (interquartile range) values, as appropriate.

Pearson’s Chi-square test was used to determine associations between socio-demographic characteristics and COVID-19 vaccine acceptability. A simple logistic regression was conducted to screen all the independent variables into the multiple logistic regression analysis (MLR). Variables with *p*<0.25 and those with evidence of predicting vaccines acceptability were included in running the MLR. The MLR was performed using the Backward LR method. Multicollinearity and interaction between the variables were checked. Assumptions of model fit were checked using Hosmer-Lemeshow tests. Overall, a two-sided *p*-value of <0.05 was considered statistically significant. The final model was presented with adjusted odds ratios (aOR), 95% confidence intervals (CI) and corresponding *p*-values.

### 2.6 Ethical consideration

Ethical approval was obtained from the College of Health Sciences Research Ethics Committee, Bayero University Kano, Nigeria, with reference number BUK/CHS/HREC/169. All procedures performed in this study involving human participants complied with the institutional and/or national research committee ethical standards and the 1964 Helsinki declaration and subsequent amendments or equivalent ethical standards. Participants were provided with detailed information about the objectives of the study, the survey and the investigators. All the participants gave their digital informed consent before participating in the study. Anonymity, confidentiality, and privacy of the study data were all guaranteed and protected throughout the study.

## 3.0 Results

### 3.1 Recruitment process

**Fig 1:**
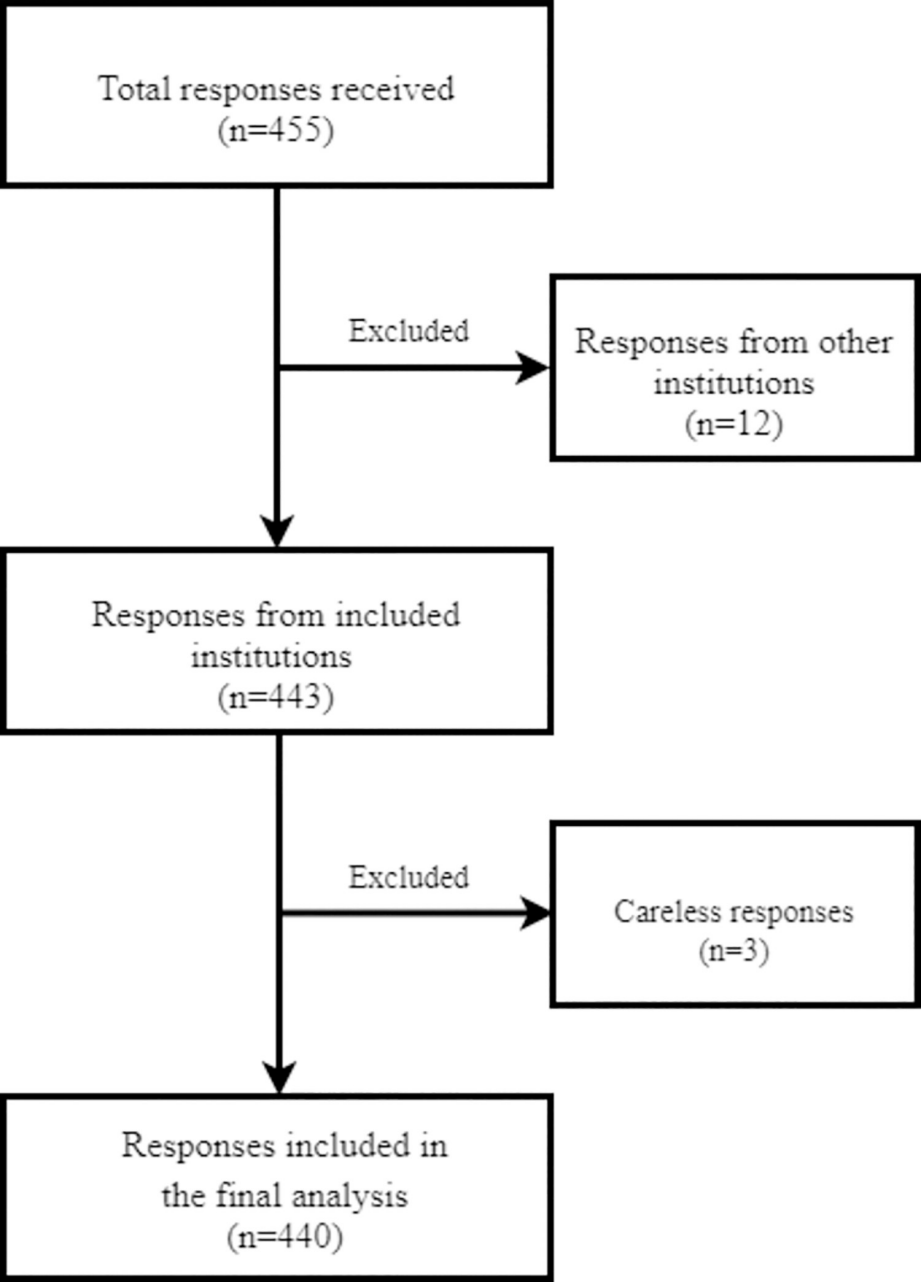
Flowchart of recruitment process of the study respondents.

A total of 455 respondents participated in the study. Of these, 12 (2.6%) respondents were excluded because they were from institutions outside the study frame. Similarly, 3 (0.7%) respondents were excluded because they were considered careless and non-objective responders as no attempt was made to answer the majority of the questions. Finally, a total of 440 valid responses (96.7% response rate) were included in the final analysis (Fig 1).

### 3.2 Socio-demographic characteristics

The median age (interquartile range, IQR) of the respondents was 23.0 (4.0) years, majority aged below 25, 296 (67.3%). The respondents were mostly females 224 (50.9%), Hausa by tribe 281 (63.9%) and not married 281 (63.9%). Most of the respondents were students of medicine 166 (37.7%) and pharmacy 133 (30.2%), mostly in their third 94 (21.8%) and fourth 88 (20.0%) years of study. Most respondents came from households with less than N50,000 ($120) monthly income 146 (33.2%). The summary of the socio-demographic characteristics of the respondents is shown in Table 1.

**Table 1:** Socio-demographic characteristics of respondents.

Variables	Total, N = 440 n (%)	Acceptance* n (%)	*p*-value^#^
**Age (years) **			
Median (IQR)	23.0 (4.0)		
Below 25	296 (67.3)	105 (35.5)	0.005
25 Above	144 (32.7)	71 (49.3)	
**Gender**			
Female	224 (50.9)	80 (35.7)	0.062
Male	216 (49.1)	96 (44.4)	
**Tribe**			
Hausa	281 (63.9)	51 (32.1)	0.011
Non-Hausa	159 (36.1)	125 (44.5)	
**Marital status**			
Single	387 (88.0)	150 (38.8)	0.151
Married	53 (12.0)	26 (49.1)	
**Course of study**			
Medicine	166 (37.7)	83 (50.0)	0.005
Pharmacy	133 (30.2)	42 (31.6)	
Nursing	103 (23.4)	42 (31.6)	
Others (E.g. Optometry)	38 (8.6)	11 (28.9)	
**Current level (L)**			
100L	72 (16.4)	21 (29.2)	0.012
200L	62 (14.1)	27 (43.5)	
300L	94 (21.8)	31 (33.0)	
400L	88 (20.0)	41 (46.6)	
500L	70 (15.9)	25 (35.7)	
600L	54 (12.3)	31 (57.4)	
**Household income (Naira) $1 = N395**			
< N50,000	146 (33.2)	55 (37.7)	0.553
N50,001—N100,000	91 (20.7)	45 (49.5)	
N100,001—N200,000	69 (15.7)	25 (36.2)	
N200,001—N300,000	42 (9.5)	14 (33.3)	
N300,001—N400,000	38 (8.6)	15 (39.5)	
N400,001 –N500,000	34 (7.7)	14 (41.2)	
> N500,000	20 (4.5)	8 (40.0)	
**Presence of chronic disease**			
No	407 (92.5)	21 (63.6)	0.004
Yes	33 (7.5)	155 (38.1)	

* = COVID-19 acceptance dichotomized as *acceptance* (yes, will take the vaccine) and *refusal* (no, will not take the vaccine)

^#^ = Chi-square test, statistical significance at *p*<0.05.

### 3.3 COVID-19 risk perception and acceptance of the vaccine

Among all the respondents, only 25 (5.7%) had previously contracted COVID-19, and 65 (14.8%) participated in COVID-19 programs. Most of the respondents had received other vaccines in the past 283 (64.3%), and only a few had previously refused a vaccine 42 (9.5%). The proportion of the respondents who accepted to take the COVID-19 vaccine was 40% (n = 176). In addition, 324 (73.6%) were willing to take the vaccine if mandated by their institution heads. Most of the respondents stated they would not recommend the COVID-19 vaccine to other people 276 (62.7%), also they were not willing to pay for the vaccine 372 (84.5) and did not trust the government regarding the vaccine 324 (73.6%). The summary of the students’ experience with COVID-19 and acceptance of the COVID-19 vaccine is shown in Table 2.

**Table 2:** COVID-19 risk perception and acceptance of the vaccine.

Variables	n (%)
**Confirmed positive for COVID-19** ^**a**^	
No	415 (94.3)
Yes	25 (5.7)
**Part of COVID-19 program**	
No	375 (85.2)
Yes	65 (14.8)
**Previously took other vaccines**	
No	157 (35.7)
Yes	283 (64.3)
**Previously refused other vaccines**	
No	398 (90.5)
Yes	42 (9.5)
**Accept to take COVID-19 vaccine**	
No	264 (60.0)
Yes	176 (40.0)
**Take COVID-19 vaccine if mandated by my heads of institution**	
No	210 (47.7)
Yes	230 (52.3)
**Recommend COVID-19 vaccine to others**	
No	276 (62.7)
Yes	164 (37.3)
**Pay for the COVID-19 vaccine if it is not free**	
No	372 (84.5)
Yes	68 (15.5)
**Trust the government on the COVID-19 vaccine**	
No	324 (73.6)
Yes	116 (26.4)

^a^ = Self-reported.

### 3.5 Factors associated with acceptance of COVID-19 vaccines

The simple logistic regression showed that factors associated with acceptance of COVID-19 vaccine include age of 25 years and above (OR, 1.77; 95% CI, 1.18–2.65; *p* = 0.006), non-Hausa tribe (OR, 1.68; 95% CI, 1.13–2.55; *p* = 0.011), fouth year students (OR, 2.12; 95% CI, 1.10–4.09; *p* = 0.025), 6th year students (OR, 3.27; 95% CI, 1.56–6.87; *p* = 0.002), having chronic disease (OR, 2.85; 95% CI, 1.36–5.95; *p* = 0.005), contracting COVID-19 (OR, 4.18; 95% CI, 1.71–10.24, *p* = 0.002), accepting to take the vaccine if directed by heads of institution (OR, 33.35; 95% CI; 18.09–61.49; *p*<0.001), willingness to pay for the vaccine if not free (OR, 23.39; 95% CI, 9.83–55.62; *p*<0.001) and trust in the government on the vaccine (OR, 50.82; 95% CI, 23.62–109.36; *p*<0.001). However, being a pharmacy student (OR, 0.46, 95% CI, 0.29–0.74; *p* = 0.001) and other health related courses (OR, 0.41; 95% CI; 0.19–0.88; *p* = 0.021) were associated with decreased odds of accepting the COVID-19 vaccines compared to studying medicine. The summary of the unadjusted factors associated with acceptance of COVID-19 vaccine are presented in Table 3.

**Table 3:** Simple logistic regression model.

Variables	OR (95% CI)	*p*-value
**Age (years)**		
Below 25	1	
25 Above	1.77 (1.18–2.65)	0.006
**Gender**		
Female	1	
Male	1.44 (0.98–2.11)	0.062
**Tribe**		
Hausa	1	
Non-Hausa	1.68 (1.13–2.55)	0.011
**Course of study**		
Medicine	1	
Pharmacy	0.46 (0.29–0.74)	0.001
Nursing	0.64 (0.39–1.05)	0.075
Others	0.41 (0.19–0.88)	0.021
**Current level**		
100L	1	
200L	1.87 (0.92–3.83)	0.085
300L	1.20 (0.61–2.33)	0.600
400L	2.12 (1.10–4.09)	0.025
500L	1.35 (0.67–2.73)	0.405
600L	3.27 (1.56–6.87)	0.002
**Presence of chronic disease**		
No	1	
Yes	2.85 (1.36–5.95)	0.005
**Confirmed positive for COVID-19** ^**a**^		
No	1	
Yes	4.18 (1.71–10.24)	0.002
**Previously took other vaccines**		
No	1	
Yes	1.33 (0.89–1.99)	0.168
**Previously refused other vaccines**		
No	1	
Yes	0.57 (0.28–1.15)	0.116
**Take COVID-19 vaccine if directed by heads of institution**		
No	1	
Yes	33.35 (18.09–61.49)	<0.001
**Pay for COVID-19 vaccine if not free**		
No	1	
Yes	23.39 (9.83–55.62)	<0.001
**Trust Government on COVID-19 vaccine**		
No	1	
Yes	50.82 (23.62–109.36)	<0.001

**Abbreviations:** OR = odds ratio; CI = confidence interval

^a^ = Self-reported.

The multivariable analyses revealed that age of 25 years and above (aOR, 2.72; 95% CI, 1.44–5.16; *p* = 0.002), accepting to take COVID-19 vaccine if directed by heads of the institution (aOR, 11.71; 95% CI,5.91–23.20; *p*<0.001), trust in the government regarding the vaccine (aOR, 20.52; 95% CI, 8.18–51.51; *p*<0.001), and willingness to pay for the vaccine if not free (aOR, 7.92; 95% CI, 2.63–23.85; *p*<0.001) were independently associated with COVID-19 vaccine acceptance. However, previous refusal to take vaccines (aOR, 0.18; 95% CI, 0.05–0.60; *p* = 0.006) was associated with decreased odds of accepting the COVID-19 vaccine. There was no multicollinearity and interaction observed in the model. The Hosmer-Lemeshow test showed a good fit model with no significance (*p* = 0.110). The classification table shows an overall accuracy of 60%. The summary of the independent predictors of acceptance of the COVID-19 vaccine among the respondents is presented in Table 4.

**Table 4:** Multivariable logistic regression model.

Variables	aOR (95% CI)	*p*-value
**Age (years)**		
Below 25	1	
25 Above	2.72 (1.44–5.16)	0.002
**Previously refused vaccines**		
No	1	
Yes	0.18 (0.05–0.60)	0.006
**Take COVID-19 vaccine if mandated by heads of institution**		
No	1	
Yes	11.71 (5.91–23.20)	<0.001
**Pay for COVID-19 vaccine if not free**		
No	1	
Yes	7.92 (2.63–23.85)	<0.001
**Trust the Government on COVID-19 vaccines**		
No	1	
Yes	20.52 (8.18–51.51)	<0.001

**Abbreviations:** aOR = adjusted odds ratio; CI = confidence interval.

## 4.0 Discussion

Our study identified the baseline prevalence of acceptance of COVID-19 vaccines among the students of health sciences in Northwest Nigeria to be low. Despite the perception of increased risk of exposure to the virus, three out of five students declined to accept the vaccine. In addition, increasing age, the influence of heads of the institution, trust in the government, and readiness to pay for the vaccine were associated with acceptance of the COVID-19 vaccine. It was interesting that contrary to the assertion that health sciences students should have a high rate of accepting the vaccine, given their predisposed medical knowledge and prospect as healthcare professionals, we instead had fewer students accepting to take the vaccines. Our findings are comparable to a recent study among undergraduate nursing students in Europe with 43.8% acceptance for COVID-19 vaccination [32]. Another study in Africa among medical students in Uganda showed far lower COVID-19 vaccine acceptability 37.3% [33]. It is worrisome that decreased COVID-19 vaccine acceptability or vaccine hesitancy is associated with increased risks of contracting the virus [34]. Vaccines’ hesitancy is a potentially major setback in the fight against the COVID-19 [35].

The socio-demographic dispositions are key determinants of embracing varying medical interventions, including age, gender, and ethnicity. In this study, students aged 25 and above were about two and a half more likely to accept COVID-19 vaccines. Increasing age in the educational systems is commonly attributed to higher knowledge and exposure to medical knowledge among health science students. In addition, older persons have higher perceived health risks, including COVID-19 infection, compared to the younger ones [36]. A study has shown increased COVID-19 vaccine acceptance among older adults [37]. Another study found a low perceived risk of infection among medical students [38]. Therefore, an evidence-based intervention must prioritize younger adults as necessary.

Despite being the major ethnic group in northern Nigeria, before adjustment, we found the Hausa tribe to be less likely to accept COVID-19 vaccines than non-Hausa counterparts, including the Yoruba and Igbo tribes. So also, the northern region has had a long history of boycotting vaccination programs, notably the oral polio vaccine, for the past two decades [39]. The lower literacy level in this region has been a subject of debate, likely attributed to the tendency to reject vaccines [40]. Higher level students have been shown to accept the vaccines because they are more likely to have come across and recognize COVID-19 patients during clinical postings and are believed to be more knowledgeable on the virus and its pathogenicity [41,42]. It has been reported that people who have come across COVID-19 related mortalities are more willing to accept vaccines [43].

In our study, those that refused other vaccines in the past were less likely to accept the COVID-19 vaccine. Previous studies have shown that individuals who previously received a vaccine against flu have a higher chance to accept to take the COVID-19 vaccine [35,44]. Our study revealed that willingness to pay for the vaccine was a significant predictor of COVID-19 vaccine acceptance. Willingness to pay for a vaccine is an indicator of the increased perceived risk of the COVID-19 infection. In a similar study in Jordan, the willingness to pay for the vaccine was a predictor of acceptance [37]. Another study reported that respondents who agreed that the pandemic was a serious public threat were willing to pay for a hypothetical vaccine [45]. Vaccination convenience regarding availability and affordability remains an essential factor to consider when investigating vaccine acceptability [46].

The present study has revealed that trusting the government was a significant predictor of COVID-19 vaccine acceptance, similar to other studies [47]. However, studies among nursing students reported the contrary [42,48]. Low confidence in politicians, lack of political consensus by the government, and mistrust of the government’s policies could potentially impact COVID-19 vaccine uptake [35]. The mistrust of the government regarding the COVID-19 vaccine revealed by the majority of the respondents (73.6%) in this research could be related to the public distrust of the government in the African region, including Nigeria, following the delay in the COVID-19 response, such as delay to implement border closure as well as allowing their relatives to return home from COVID-19 high-risk countries without proper preventive measures among others [49].

We acknowledge some limitations to our study findings. First, potential targets who did not respond to the survey might have been hesitant about the vaccine, which could underestimate the actual prevalence of vaccine acceptability due to bias. The study was also limited by the convenience sampling and snowball sampling technique employed due to circumstances associated with the pandemic. Thus, the authors couldn’t control the recruitment process but the people addressed in the initial rounds. Therefore, the findings from the data collected from four institutions in three states out of seven states of Northwestern Nigeria may not be generalizable. Despite these limitations, the study contributes significantly to understanding COVID-19 vaccine acceptability and its determinants, particularly among university health science students in Northwest Nigeria.

## 5.0 Conclusions

The prevalence of COVID-19 vaccine acceptance among the students of the healthcare profession in Northwest Nigeria was low. Older age, the influence of heads of institutions, readiness to pay for the vaccines and trust in the government were associated with vaccine acceptance. Since vaccination appears to be an essential preventive measure to curb the menace of COVID-19, factors relating to low vaccine acceptance among vulnerable groups like young adults need to be urgently addressed by inclusive public health strategies.

## Supporting information

S1 File STROBES checklist followed in reporting the study. (PDF)

S2 File Study survey tool for data collection. (PDF)
