# Supplementary material for: Factors associated with acceptance of COVID-19 vaccine among University health sciences students in Northwest Nigeria
Source: PLoS One. 2021 Nov 29;16(11):e0260672. doi: 10.1371/journal.pone.0260672 (PMC8629299; doi:10.1371/journal.pone.0260672)
Supplement: S2 File — (PDF) [file pone.0260672.s002.pdf]

QUESTIONNAIRE

COVID-19 vaccine acceptance among university health science students in Northwest Nigeria

Dear respondents,

Thank you for participating in this COVID-19 vaccine study. This study aims to find out the level of your acceptance to be vaccinated against COVID-19. Be rest assured that all your provided information will be treated under the General Data Protection provisions.

Consent

• I agree to participate in this survey.
• I understand that all data will be treated confidentially by the researcher.
• I can withdraw at any time without giving a reason

Validation

Are you a university student of health sciences in Northwest Nigeria?

Section I: Socio-demographic characteristics

1. Gender: [a]. Female [b]. Male
2. Age (Years): __________
3. State of Origin: ________
4. Religion: [a]. Islam [b]. Christianity
5. Tribe: [a]. Hausa [b]. Igbo [c]. Yoruba [d]. Others
6. Marital status: [a]. Single [b]. Married [c]. Widowed [d]. Divorced [e]. Seperated
7. Course of study: [a]. Medicine [b]. Pharmacy [c]. Nursing [d]. Others
8. Family income (monthly): [a]. <N50K [b]. 50K-100K [c]. 100K-200K [d]. 200K-300K [e]. 300K-400K [f]. 400K-500K [g]. >500K
9. Do you have any chronic disease? [a]. Yes [b]. No

Section II: Risk perception of COVID-19

10. Have you ever been tested for COVID-19? [a]. Yes [b]. No
11. Have you ever been confirmed infected with COVID-19? [a]. Yes [b]. No
12. Are you worried about getting infected with COVID-19? [a]. Yes [b]. No [c]. Not sure
13. Have you ever been part of any COVID-19 response team or program? [a]. Yes [b]. No

14. Have you ever been vaccinated in the past? [a]. Yes [b]. No
15. Have you ever refused vaccination in the past? [a]. Yes [b]. No

Section III: Acceptability of COVID-19

16. Are you aware that the Nigerian Government is planning to vaccinate all citizens against COVID-19? [a]. Yes [b]. No [c]. Not sure
17. Are you willing to be vaccinated against COVID-19? [a]. Yes [b]. No [c]. Not sure
18. Do you agree that the COVID-19 vaccine protects against the virus? [a]. Yes [b]. No [c]. Not sure
19. Do you trust the Government on the COVID-19 vaccine? [a]. Yes [b]. No [c]. Not sure
20. Will you take the COVID-19 vaccine if mandated by the heads of your institution? [a]. Yes [b]. No [c]. Not sure
21. Will you recommend the COVID-19 vaccine to others? [a]. Yes [b]. No [c]. Not sure
22. Will you pay for the COVID-19 vaccination if it is not free? [a]. Yes [b]. No [c]. Not sure

ONLINE GOOGLE FORM QUESTIONAIRE

Link here: https://forms.gle/Vso4gRxSFrsB9UoF7
